# Efficacy and safety of rapid intermittent correction compared with slow continuous correction with hypertonic saline in patients with moderately severe or severe symptomatic hyponatremia: study protocol for a randomized controlled trial (SALSA trial)

**DOI:** 10.1186/s13063-017-1865-z

**Published:** 2017-03-29

**Authors:** Anna Lee, You Hwan Jo, Kyuseok Kim, Soyeon Ahn, Yun Kyu Oh, Huijai Lee, Jonghwan Shin, Ho Jun Chin, Ki Young Na, Jung Bok Lee, Seon Ha Baek, Sejoong Kim

**Affiliations:** 1 Department of Internal Medicine, Seoul National University Bundang Hospital, Seongnam, Republic of Korea; 2 Department of Emergency Medicine, Seoul National University Bundang Hospital, Seongnam, Republic of Korea; 3 Department of Medical Research Collaborating Center, Seoul National University Bundang Hospital, Seongnam, Republic of Korea; 4 Department of Internal Medicine, Seoul National University Boramae Hospital, Seoul, Republic of Korea; 5 Department of Emergency Medicine, Seoul National University Boramae Hospital, Seoul, Republic of Korea; 6 Clinical Epidemiology and Biostatistics, University of Ulsan College of Medicine, Asan Medical Center, Seoul, Republic of Korea; 7 Department of Internal Medicine, Hallym University Dongtan Sacred Heart Hospital, Hwaseong-si, Republic of Korea

**Keywords:** Hyponatremia, Hypertonic saline, Treatment, Osmotic demyelination syndrome

## Abstract

**Background:**

Hyponatremia is the most common electrolyte imbalance encountered in clinical practice, associated with increased mortality and length of hospital stay. However, no high-quality evidence regarding whether hypertonic saline is best administered as a continuous infusion or a bolus injection has been found to date. Therefore, in the current study, we will evaluate the efficacy and safety of rapid intermittent correction compared with slow continuous correction with hypertonic saline in patients with moderately severe or severe symptomatic hyponatremia.

**Methods/design:**

This is a prospective, investigator-initiated, multicenter, open-label, randomized controlled study with two experimental therapy groups. A total of 178 patients with severe symptomatic hyponatremia will be enrolled and randomly assigned to receive either rapid intermittent bolus or slow continuous infusion management with hypertonic saline. The primary outcome is the incidence of overcorrection at any given period over 2 days. The secondary outcomes will include the efficacy and safety of two other approaches to the treatment of hyponatremia with 3% hypertonic saline.

**Discussion:**

This is the first clinical trial to investigate the efficacy and safety of rapid intermittent correction compared with slow continuous correction with hypertonic saline in patients with moderately severe or severe hyponatremia.

**Trial registration:**

ClinicalTrials.gov, identifier number: NCT02887469. Registered on 1 August 2016.

**Electronic supplementary material:**

The online version of this article (doi:10.1186/s13063-017-1865-z) contains supplementary material, which is available to authorized users.

## Background

Hyponatremia is the most common electrolyte imbalance encountered in clinical practice, and is associated with increased mortality and length of hospital stay [1]. The symptoms related to hyponatremia span a broad range from moderately severe (cognitive test abnormalities, gait impairment) to severe or life-threatening (hypoxemia, coma, and epilepsy) [2–5]. Treatment strategies for hyponatremia differ according to the severity of clinical symptoms and duration of hyponatremia [6]. The extent and rate of increase in serum sodium (sNa) levels during treatment are crucial. Overcorrection of chronic hyponatremia may result in osmotic demyelination syndrome (ODS), whereas undercorrection may be insufficient to prevent its life-threatening manifestations [6–9].

Several methods for continuous infusion of hypertonic saline have been used to guide the rate of fluid administration to achieve the required sNa target [10, 11]. The increase in sNa should be limited to 8–10 mmol/L within the first 24 h and 18 mmol/L within 48 h [11, 12]. As these methods were based on static models of a dynamic clinical situation, they had a bias toward overcorrection of hyponatremia [1, 13–15].

Recent guidelines have recommended prompt infusion of 100–150 mL of 3% saline over 10–20 min to increase the sNa by 4–6 mmol/L during the first hour. A pragmatic approach to treatment with hypertonic fluid is to use small, fixed boluses to achieve controlled incremental increases in sNa [1, 13–17]. However, this recommendation was the result of small randomized trials [18], case reports with small numbers of patients [14, 19], and expert opinion [20–23]. No high-quality evidence has been reported regarding whether hypertonic saline is best administered as a continuous infusion (preferred by most) or as a rapid intermittent bolus injection. The purpose of the present study was to investigate the efficacy and safety of rapid intermittent correction compared with slow continuous correction with hypertonic saline in patients with symptomatic severe hyponatremia. Furthermore, the study will examine prevailing conceptions regarding the optimal treatment of severe symptomatic hyponatremia.

## Methods/design

### Hypothesis

The use of intermittent bolus therapy with 3% sodium chloride will prevent and/or ameliorate ODS in patients with hyponatremia treated with hypertonic saline. Compared to the use of standard slow continuous infusion with 3% hypertonic saline, the incidence of overcorrection will be lower in patients with moderately severe or severe hyponatremia.

### Study design

**Fig. 1:**
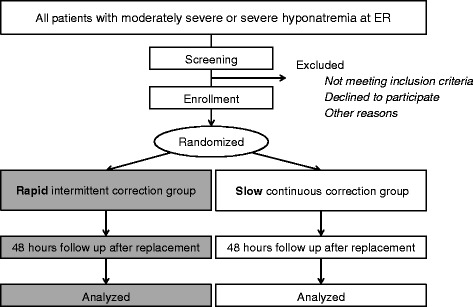
Study algorithm. *ER* emergency room

**Fig. 2:**
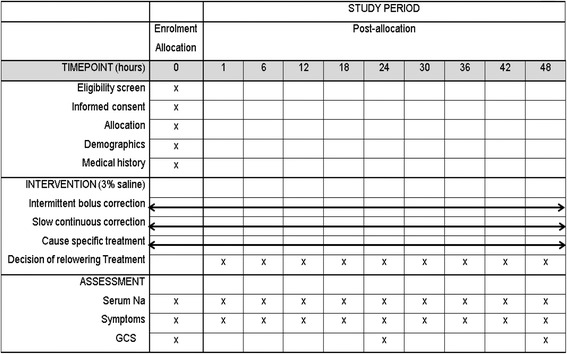
Schedule of enrollment, interventions, and assessments according to the Standard Protocol Items: Recommendations for Interventional Trials (SPIRIT) guideline. *GCS* Glasgow Coma Scale

**Fig. 3:**
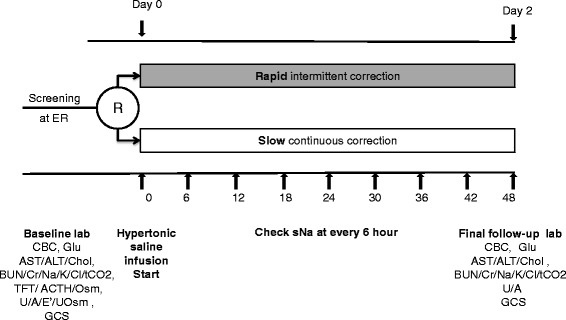
Study schedule. *R* randomization, *ER* emergency room, *CBC* complete blood count, *Glu* glucose, *AST* aspartate aminotransferase, *ALT* alanine aminotransferase, *Chol* cholesterol, *BUN* blood urea nitrogen, *Cr* creatinine, *tCO* *2* total CO2, *TFT* thyroid function test, *ACTH* rapid adrenocorticotropic hormone, *Osm* Osmolality, *U/A* urinalysis, *E'* electrolyte, *UOsm* Urine osmolality, *GCS* Glasgow Coma Scale

This study is a prospective, investigator-initiated, multicenter, open-label, randomized controlled study with two experimental therapy groups. We have followed the Standard Protocol Items: Recommendations for Interventional Trials (SPIRIT) 2013 Statement which defines standard protocol items for clinical trials [24] (see Additional file 1). The study algorithm is depicted in Fig. 1 and the SPIRIT and study schedule are given in Figs. 2 and 3. After enrollment, clinical follow-up will be performed 2 days after treatment with 3% hypertonic saline.

### Study participants and measurements

All participants will be selected from among patients visiting the emergency rooms of two general hospitals in Korea (Seoul National University Bundang Hospital, Seoul National University Boramae Medical Center). Patients aged over 18 years who have moderately severe to severe symptoms and glucose-corrected sNa [25] ≤125 mmol/L on the first blood test will be screened for study participation. Moderate symptoms include nausea, headache, drowsiness, general weakness, and malaise [1, 26, 27]. Severe symptoms include vomiting, stupor, seizure, and coma (Glasgow Coma Scale (GCS)).

The following assessments will be performed during the initial emergency room encounter: (1) completion of questionnaire on medical and drug history including the use of antihypertensive medication, diuretics, nonsteroidal anti-inflammatory drugs, selective serotonin reuptake inhibitors, antiepileptic drugs, steroids, and synthyroid, (2) physical examination of all body systems, (3) measurement of height and weight, (4) blood pressure and pulse rate measurement, and (5) assessment of psychological status. Participants, who meet all of the inclusion criteria, do not require exclusion on the basis of the exclusion criteria, and provide written informed consent will be eligible for this study (Table 1).

**Table 1:** Inclusion and exclusion criteria

Inclusion criteria	Exclusion criteria
18 years and older	Pseudo-hyponatremia (serum osmolality >275 mOsm/kg)
	Primary polydipsia (urine osmolality ≤100 mOsm/kg)
	Glucose-corrected serum sodium >125 mmol/L
	Initial arterial hypotension (SBP <90 mmHg and MAP <70 mmHg)
Glucose-corrected serum sodium ≤125 mmol/L	Anuria or urinary outlet obstruction
	Decompensated LC (LC with ascites or diuretics use or history of hepatic encephalopathy and varices)
	Bilirubin >2 mg/dL, transaminase levels >2.5 times the upper limit of normal)
Symptomatic patients	Uncontrolled diabetes mellitus (HbA1C >9%)
Moderately severe:	Women who are pregnant or breastfeeding
nausea without vomiting, drowsiness, headache, general weakness, malaise	Within the 3 months prior to randomization, history of cardiac surgery excluding PCA, acute myocardial infarction, sustained ventricular tachycardia, ventricular fibrillation, acute coronary syndrome, cerebrovascular trauma, and increased intracranial pressure
Severe:	
vomiting, stupor, seizures, coma (GCS ≤8)	

*GCS* Glasgow Coma Scale, *SBP* systolic blood pressure, *MAP* mean arterial pressure, *LC* liver cirrhosis, *HbA1C* glycosylated hemoglobin, *PCA* percutaneous coronary angioplasty

Serum creatinine (SCr) will be measured using the isotope dilution mass spectrometry-traceable method with a Toshiba TBA 200FR Analyzer (Toshiba, Tokyo, Japan). The estimated glomerular filtration rate (eGFR) will be calculated using the Chronic Kidney Disease Epidemiology Collaboration (CKD EPI) equation [28].

### Randomization

A research coordinator will perform the randomization. A list of random numbers will be generated by an independent statistician. Eligible participants will be randomly assigned 1:1 to either the slow continuous correction group or the rapid intermittent correction group in accordance with the predefined randomization list with a block size of 2. Randomization will be stratified based on the symptom severity related to hyponatremia (moderately severe or severe).

### Physician’s practical treatment guidelines according to the sNa level

The principal purpose of this study will be to compare the rapid intermittent correction group to the slow continuous correction group [29]. According to a review of the medical literature on the treatment of hyponatremia, treatment with hypertonic (3%) saline is recommended in cases of severe symptomatic hyponatremia [1, 15, 16]. Therefore, the rate of infusion of 3% saline will be stratified based on the symptom severity in each group. After randomization, the participants will undergo either rapid intermittent correction or slow continuous correction of hyponatremia for 48 h. Treatment in the intermittent bolus group will comply with European guidelines published in 2014, and the slow continuous infusion will be guided by widely accepted methods [1, 13–16]. The treatment goals are to raise the sNa level by 5–9 mmol/L from the initial sNa and to achieve symptom relief within the first 24 h, and to raise the sNa level by 10–17 mmol/L from the initial sNa or ≤130 mmol/L, and to achieve symptom relief during the 48 h [10–12]. We will also check for overcorrection at every sample time point and conduct relowering treatment. Relowering treatment is performed as below if the achieved sNa level is ≥10 mmol/L at any sample time point within the first 24 h or the achieved sNa level is ≥18 mmol/L at any sample time point within 48 h [1, 13–16]. The treatment goal, relowering treatment strategy, and cause-specific treatment of hyponatremia will be applied to each group equally.

## Rapid intermittent bolus group (Fig. 4a)

### First 24 hours

In the case of moderately severe symptoms, the use of intravenous infusion of 2 mL/kg 3% hypertonic saline over 20 min (100 mL, 3% saline for unknown body weight) is recommended. In cases of severe symptoms, it is recommended to perform intravenous infusion of 4 mL/kg 3% hypertonic saline over 40 min (200 mL, 3% saline for unknown body weight). After the initial treatment, repeated infusion of 2 mL/kg 3% hypertonic saline over 20 min at every sample time point (at 1, 6, 12, 18, and 24 h) until the observation of a sNa level increase of 5–9 mmol/L from the initial sNa and symptom relief is recommended.

**Fig. 4:**
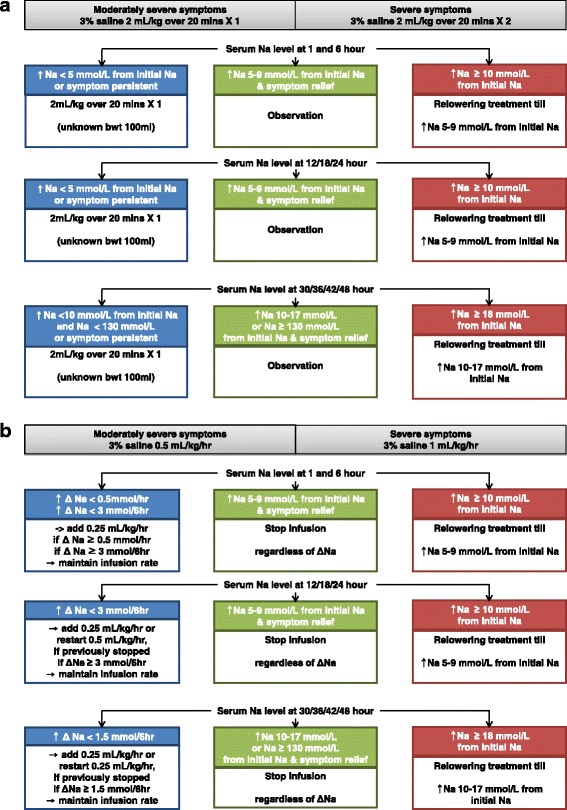
Treatment. **a** Rapid intermittent correction with hypertonic saline in patients with moderately severe or severe symptomatic hyponatremia. *min* minute, *Na* sodium, *bwt* body weight. ↑, increase. **b** Slow continuous correction with hypertonic saline in patients with moderately severe or severe symptomatic hyponatremia. *hr* hour, *Na* sodium, *bwt* body weight. ↑, increase; Δ, delta – change in amount

### From 24 to 48 hours

In both moderately severe and severe cases of hyponatremia, repeated infusion of 2 mL/kg 3% hypertonic saline over 20 min at every sample time point (at 30, 36, 42, and 48 h) is recommended until the sNa level increases to 10–17 mmol/L from the initial sNa or until the sNa reaches 130 mmol/L, and until the symptoms improve.

## Slow continuous correction group (Fig. 4b)

### First 24 hours

In the case of moderately severe symptoms, it is recommended to infuse 3% hypertonic saline at a rate of 0.5 mL/kg/h (25 mL/h, 3% saline for unknown body weight). In cases of severe symptoms, it is recommended to begin an infusion of 3% hypertonic saline at a rate of 1 mL/kg/h (50 mL/h, 3% saline for unknown body weight). After the initial treatment, the infusion protocol will be modified as below according to the sNa level at every sample time point (at 1, 6, 12, 18, and 24 h). If the sNa level increases to 5–9 mmol/L above the initial sNa level, accompanied by symptom relief, the 3% saline infusion should be discontinued regardless of changes in the sNa level. If the sNa level increases at a rate of under 0.5 mmol/h or 3 mmol/6 h, it is recommended to add 3% hypertonic saline infusion at a rate of 0.25 mL/kg/h to the previous infusion rate or to restart the infusion at 0.5 mL/kg/h if it was previously discontinued. If the sNa level demonstrates increases of over 0.5 mmol/h or 3 mmol/6 h, the rate of 3% hypertonic saline infusion should be maintained.

### From 24 to 48 hours

The infusion protocol will be modified as below according to the sNa levels at every sample time point (at 30, 36, 42, and 48 h). If the Na level increases to 10–17 mmol/L above the initial sNa level or sNa reaches 130 mmol/L, and symptom relief is observed, the 3% hypertonic saline infusion should be discontinued regardless of changes in the sNa level during the previous 6 h. If the Na level increase is less than 1.5 mmol/6 h, it is recommended to add 3% hypertonic saline infusion at a rate of 0.25 mL/kg/h to the previous infusion rate or to restart the infusion at a rate of 0.25 mL/kg/h if it was previously discontinued. If sNa levels increase at a rate of over 1.5 mmol/6 h, the 3% saline infusion rate should be maintained.

### Outcome measures

The primary outcome is the incidence of overcorrection, a surrogate marker of ODS, at any given period, defined as follows: increase in sNa level by >12 mmol/L within the first 24 h or increase in sNa level by >18 mmol/L within 48 h. The secondary outcomes will include efficacy and safety; changes in symptoms between pretreatment and 24 and 48 h after treatment with 3% hypertonic saline; time from treatment initiation to an increase in sNa ≥5 mmol/L; time from treatment initiation to the achievement of a sNa >130 mmol/L; incidence of target correction rate, defined as an achieved sNa <10 mmol/L within 24 h and a sNa <18 mmol/L within 48 h; length of hospital stay; incidence of additional treatment; incidence of ODS confirmed by the *International Classification of Disease, 10th Revision (ICD-10)* code or magnetic resonance imaging (MRI); incidence of relowering treatment; and change in GCS score between pretreatment and 24 and 48 h after 3% hypertonic saline treatment.

### Clinical and laboratory evaluations

The physical examination, laboratory evaluations, and medication reviews will be conducted before treatment; the laboratory evaluations will include a complete blood count, serum electrolytes, blood urea nitrogen, creatinine, calcium, phosphorous, protein, albumin, and glucose; a liver function test; a thyroid function test (TFT); rapid adrenocorticotropic hormone (ACTH) test (basal cortisol, ACTH, cortisol at 30 min and 60 min), serum osmolality; urinalysis, urine electrolytes, urine osmolality. sNa levels will be measured every 6 h for 2 days [30]. The GCS score will be assessed before treatment and at 24 and 48 h of treatment. All types and volumes of administered fluid during 48 h will be monitored.

### Safety issues

All adverse events related to overcorrection will be recorded and followed up during the study period or until resolution during treatment with 3% hypertonic saline. Any serious adverse events will be reported to investigators and the Ethics Committee. We will check for overcorrection at every sample time point and conduct relowering treatment as needed. Relowering treatment is performed as follows if the achieved sNa level is ≥10 mmol/L at any sample time point within the first 24 h or if the achieved sNa level is ≥18 mmol/L at any sample time point within 48 h: discontinuing ongoing active treatment; initiating infusion of 10 mL/kg of 5% dextrose over 1 h; and/or adding desmopressin 2 mcg [1].

### Sample size calculation

In the medical literature, ODS is a critically important outcome, but the incidence of ODS is very low [1, 15, 31]. Therefore, as a surrogate marker of ODS, the rate of overcorrection >12 mmol/L within 24 h or >18 mmol/L within 48 h was calculated for the primary study outcome. A previous study reported that the overcorrection rate was 10–16% with slow continuous correction of hyponatremia [10]. However, there was insufficient information for estimating the rate of overcorrection with intermittent bolus infusion. Based on clinical practice and experience, we estimated that the overcorrection rate was 32% with slow continuous correction during a recent 1-year period. We therefore expect that the rate of overcorrection will be 5% in patients treated with rapid intermittent correction and the rate of overcorrection with slow continuous correction could be as high as 20%. We calculated the required sample size for an estimated dropout rate of 15%, a two-sided level of significance of *α* = 5%, a power of 80%, and found that 89 participants will be required in each group to find a significant difference using a *χ* ^*2*^ test. A total of 178 participants will be included in the analysis. We have considered one interim analysis at the time that a half of subjects complete the study. The O’Brien-Fleming’s alpha spending function will be used to test the first interim and final analysis of primary outcome.

### Statistical analyses

Statistical analyses will be performed on both per-protocol (PP) and intention-to treat (ITT) bases. For the PP analysis, all participants who completed the study will be included to evaluate the primary and secondary outcomes. For the ITT analysis, all participants who were enrolled and randomized to one of the two groups will be included. The baseline characteristics and laboratory data will be presented as means and standard deviations for continuous variables and as frequencies and percentages for categorical variables.

The incidence of overcorrection, ODS, relowering treatment, and target correction rate (achieved sNa <10 mmol/L within the first 24 h and sNa <18 mmol/L within 48 h) will be compared between the two groups using a *χ* ^*2*^ test and Fisher’s exact test. In interim analysis, if the *P* value < 0.003 then we would stop the study early for significance of outcome. If not, we would recruit another half of subjects. The differences in changes in symptoms and GCS scores between pretreatment and 24 and 48 h, time to reach target sNa, time to achieved sNa ≥5 mmol/L for the first time, and length of hospital stay will be analyzed using Student’s *t* test or the Mann-Whitney *U* test. A value of *P* < 0.05 will be considered statistically significant. All analyses will be performed using SPSS Statistics software V21.0 (IBM Corporation, Armonk, NY, USA).

### Data and safety monitoring

The paper data collection sheets and signed informed consents will be stored in a locked cabinet, and the electronic data base will be stored on password-protected secure severs. Any unanticipated adverse events that occur during the study will be reported to the Institutional Review Board in accordance with the procedures of Seoul National University Bundang Hospital, Korea. For any revised study procedure, the modification will be submitted to the Seoul National University Bundang Hospital Institutional Review Board for approval and to ClinicalTrials.gov. The data will be kept confidential with only limited access to research investigators.

## Discussion

Recently published guidelines recommended the prompt infusion of small, fixed boluses of hypertonic saline in patients with symptomatic hyponatremia [1, 15, 16]. This regimen leads to abrupt incremental increases in sNa levels and improvement of hyponatremia-related symptoms [1, 15, 16]. However, safety issues regarding rapid correction with hypertonic saline have not been evaluated. No large-scale randomized controlled trials to evaluate and compare the efficacy and safety of rapid and slow correction with hypertonic saline have been performed. This is the first clinical trial to provide physicians with quality evidence regarding treatment with hypertonic saline in patients with moderately severe or severe hyponatremia. In addition, a protocolized approach, such as that of this study, may be extended to the management of other electrolyte disorders.

In the development of this protocol, we complied with recent guidelines, particularly in determining the target sNa levels, in the method of infusion of 3% hypertonic saline for the rapid correction group, and in the classification of symptoms of hyponatremia [1, 15, 16]. Recently published guidelines recommended prompt intravenous infusion of 100–150 mL 3% hypertonic saline over 10–20 min, repeated once or twice as needed, in the initial treatment of severe hyponatremia [1, 15, 16]. We adopted a weight-based approach (2 mL/kg) rather than the fixed 100–150-mL infusion volumes of 3% hypertonic saline in this study because Koreans tend to have smaller physiques than Westerners. Recent guidelines recommended discontinuing the infusion of hypertonic saline in cases of improvement of symptoms after a 5-mmol/L increase in sNa and initiating a diagnosis-specific treatment [1]. Our planned approach to decreases or no change in sNa levels during the diagnosis of hyponatremia and the initiation of cause specific treatment was to repeat a bolus infusion every 6 h until sNa incrementally increases to the target level and symptom relief were observed. This approach is different from the protocols recommended by recent guidelines [1]. In the slow continuous correction group, we adopted a 3% hypertonic saline infusion rate of 0.5–1 mL/kg/h, a widely known treatment approach [10, 11], according to symptom severity. Recent guidelines define indicative symptoms of cerebral edema as severe or moderate symptoms that can be attributed to hyponatremia [1]. In our study, general weakness and malaise, usually considered nonspecific, were included as moderate symptoms of hyponatremia. Our rationale for including these as symptoms of hyponatremia are as follows: first, patients start complaining of nausea and malaise as sNa concentration falls acutely below 125 mmol/L [27]. Malaise is thus a result of hyponatremia and is as significant as nausea. Second, patients with symptomatic hyponatremia, including weakness and malaise, should be treated with hypertonic saline [26]. As this clinical trial aims to compare the efficacy and safety of rapid versus slow hypertonic saline administration in the correction of hyponatremia, we included general weakness and malaise as hyponatremia-defining symptoms.

A previous study reported that the overcorrection rate was 10–16% in slow continuous correction of hyponatremia [10]. According to unpublished retrospective data in our hospital, overcorrection in the slow continuous correction group occurred in approximately 32% out of 129 hyponatremic patients during a recent 1-year period. Although Moritz et al. reported that there is little or no risk of inadvertent overcorrection [20], data on the rapid intermittent correction regimen were unavailable. We therefore expect that the overcorrection rate will be 5% in patients treated with rapid intermittent correction, and the overcorrection rate in slow continuous correction could be as high as 20%. For this reason, we will plan to perform an interim analysis, which will be conducted after a number of patients equal to half the estimated total sample size have been enrolled. No previous studies addressing differences in efficacy between rapid intermittent correction groups and slow continuous correction groups in each stratification group have been reported. In the interim analysis, we will also examine the ratio of stratification (moderately severe versus severe) for each experimental therapy group and analyze the efficacy of rapid intermittent correction compared to slow continuous correction for each level of stratification. After the interim analysis, the total study population of 178 patients will be adjusted to the ratio of stratification in the experimental therapy groups. In the interim analysis, if the *P* value < 0.003 then we will stop the study early for significance of outcome. If not, we will recruit another half of subjects.

We will also include the participants with chronic hyponatremia. It is well-known that using a threshold of 48 h is frequently applied to distinguish “acute” from “chronic” hyponatremia [1]. Because not all patients have sNa measured within 48 h prior to visiting the emergency room. Therefore, it is practically impossible to exclude the patients with chronic hyponatremia based on these definitions. Second, the most important factor in determining treatment of hyponatremia is the presence of symptoms, irrespective of biochemical degree or timing (acute versus chronic) of hyponatremia [1]. We will enroll the patients with moderately severe to severe symptoms who need to be treated. In other words, even if the patients belong to the chronic hyponatremia group, the patients with symptomatic hyponatremia need to be treated. Therefore, we thought that including the patients with chronic hyponatremia is reasonable and inevitable.

We will use the overcorrection rate as a primary outcome. ODS is a serious complication, which should be prevented for patient safety, and it is one of the critical outcomes in the management of hyponatremia, but it is difficult to diagnose and very uncommon [1, 31]. Several studies have reported that the rate of overcorrection is strongly correlated with ODS, and is highly sensitive for predicting ODS. In terms of safety issues, the overcorrection rate might be a good laboratory outcome in our trial [1, 15, 32, 33].

This trial may have some limitations. We will enroll patients who visit the emergency room in general hospitals; therefore, patients with hospital-acquired hyponatremia will be excluded. Second, various situations may affect protocol violations, considering that on-duty physicians and nurses may work in shifts. For this, reason we made both protocols as simple as possible.

In summary, the SALSA study is the first prospective, multicenter, randomized, open-label, controlled clinical trial to evaluate the clinical usefulness of rapid intermittent correction management in patients with moderately severe or severe hyponatremia. The aim of this study is to evaluate the efficacy and safety of rapid bolus correction compared with slow continuous correction of hyponatremia.

### Trial status

This trial started recruiting in August 2016. Recruitment is expected to conclude by July 2018.

## Additional file

Additional file 1: SPIRIT 2013 Checklist: recommended items to address in a clinical protocol and related documents. (DOC 122 kb)

## Abbreviations

ACTH: Rapid adrenocorticotropic hormone
CKD EPI: Chronic Kidney Disease Epidemiology
eGFR: Estimated glomerular filtration
GCS: Glasgow Coma Scale
ICD: *International Classification of Diseases*
ITT: Intention-to-treat
MRI: Magnetic resonance imaging
ODS: Osmotic demyelination syndrome
PP: Per-protocol
SCr: Serum creatinine
sNa: Serum sodium
SPIRIT: Standard Protocol Items: Recommendations for Interventional Trials
TFT: Thyroid function test
